# Identification and control of an isolated, but intense focus of lymphatic filariasis on Satawal Island, Federated States of Micronesia, in 2003

**DOI:** 10.1186/s41182-017-0050-0

**Published:** 2017-06-09

**Authors:** Moses Pretrick, Wayne Melrose, Jean-Paul Chaine, Deon Canyon, Jaime Carron, Patricia M. Graves, Richard S. Bradbury

**Affiliations:** 1 Department of Health & Social Affairs, Government of the Federated States of Micronesia, Palikir, Federated States of Micronesia; 2 College of Public Health, Medical and Veterinary Sciences, Division of Tropical Health and Medicine, James Cook University, Townsville, Queensland Australia; 3 Pacific Island Health Officers Association, Honolulu, HI USA; 4 Daniel K. Inouye Asia Pacific Center for Security Studies, Honolulu, HI USA; 5 School of Medical and Applied Sciences, Central Queensland University, Rockhampton, Queensland Australia

**Keywords:** Filariasis, Lymphatic, *Wuchereria*, Elephantiasis, Hydrocoele, Entomology, Mosquitoes, Micronesia

## Abstract

**Background:**

There is very limited data available on the prevalence of Bancroftian filariasis in the Federated States of Micronesia (FSM). Considerable attempts to eliminate the disease had occurred in the Pacific region by the year 2003, and the prevalence in FSM was thought to be sufficiently low that the region was considered non-endemic. However, a survey conducted in 2003 on an isolated atoll of FSM, Satawal Island, challenged that assumption.

**Methods:**

Participants on Satawal Island were recruited and their blood tested for *Wuchereria bancrofti* antigen by the filariasis immunochromatographic test (ICT) card and circulating microfilaria by Knott’s concentration technique. A survey for active cases of lymphoedema, elephantiasis and hydrocoele was performed and mosquitoes were trapped and dissected to detect larvae of *W. bancrofti*.

**Results:**

A total of 104 males and 149 females from early teens to mid-80s were tested. Men had a significantly higher prevalence of infection than women in both the ICT test (53 vs 28%; *p* < 0.001) and by Knott’s concentration results (37 vs 11%; *p* < 0.001). Microfilaria prevalence was higher in subjects ≤25 than in those >25 years of age. All persons sampled were treated for LF. No cases of elephantiasis or hydrocoele were detected. No *Aedes* dissected were positive but three of nine culicine mosquitoes were positive for L1–3 larval stages of *W. bancrofti* by microscopy. In depth interviews were conducted with residents and chiefs.

**Conclusions:**

This survey showed that even in regions thought to be close to elimination, isolated high intensity foci of lymphatic filariasis may occur. These need to be recognized and control measures instituted such as local MDA as in the current study.

## Background

The Pacific Programme to Eliminate Lymphatic Filariasis (PacELF), launched by WHO in 1999 [1], has made great progress toward the elimination of this disease throughout the Pacific. However, due to its great geographic expanse and limited population in many separate islands, unique epidemiological challenges have been faced in this region. Continued vigilance following elimination of lymphatic filariasis (LF) as a public health problem is warranted to avoid the possibility of re-introduction of the disease in isolated regions. Just such a situation occurred on Satawal Island in the Federated States of Micronesia (FSM) in 2003, the investigation of which is discussed in this paper.

There is very limited recent data available in the literature regarding the relative prevalence of filariasis in the Pacific region of Micronesia. FSM is the largest nation in that region, with a population of 107,008 people in the year 2000 census, a land area of 701 km^2^ and population density of 161 people per square kilometre [1]. FSM is a US-associated state consisting of four states, each consisting of several islands: Chuuk, Pohnpei, Yap and Kosrae, and many thousands of islands. The islands of FSM continue to be classified as partially endemic for Bancroftian filariasis by the WHO [2]. The predominant vectors in the region are mosquitoes of the genus *Culex* [3].

Historical blood surveys carried out during the Second World War (1943) in Yap, Chuuk and Ponape found *Wuchereria bancrofti* prevalence rates of 12.6, 22.5 and 3.2%, respectively [1]. Following these, a survey carried out on two islands in Chuuk state in the early 1980s showed a similar Bancroftian filariasis prevalence of 7.9% [4]. By the early 1990s, a survey of nine islands found a prevalence of 2.6%, though prevalence in three isolated villages remained at 7–10% [5]. A convenience sample survey carried out between 1999 and 2001 in Chuuk and Yap found only five of 2392 (0.2%) participant samples positive for *W. bancrofti* using the filarial antigen immunochromatographic card test (ICT) method [1]. At this time, the overall prevalence of LF in FSM was considered to be sufficiently negligible that the area was considered non-endemic [6].

Mass drug administration (MDA) to control filariasis had been performed several times in FSM prior to this study [1]. A diethylcarbamazine (DEC) MDA in 1956 dropped the filariasis prevalence from 22.1 to 2.9% of the FSM population. A total of 865 people on four islands of Yap state were treated with DEC in 1974. FSM was classified as partially endemic at the start of PacELF in 1999, but no MDA was started, perhaps due to skepticism about the continuation of transmission. During the early 2000s, a filariasis antigen survey using ICT cards was conducted throughout FSM. All surveys found <1% antigen prevalence except in 2002 in Tamatam and Satawal Island, where 19 of 50 children (38%) aged 8–15 years were positive [1].

In February 2003, a multi-disciplinary team from FSM and Yap Departments of Health and the WHO Collaborating Center for Control of Lymphatic Filariasis, School of Public Health and Tropical Medicine, James Cook University, traveled to investigate this unexpected focus of LF infection, conduct a more intensive survey and produce a report for the FSM health authorities which is now being published to allow the broader scientific and medical community another source of access to further historical survey data regarding parasitic infection in the FSM and to present important evidence of the highly focal nature of filarial infection in Pacific Island nations.

Satawal Island is the eastern-most island in Yap state. The total land area is 1.3 km^2^ and is thickly wooded with coconut and breadfruit trees. The total population varies between 450 and 650 people due irregular exchange of residents to and from Satawalese communities elsewhere, mainly on the island of Chuuk. Literacy is above 90%, and English is commonly spoken. The population live in closely spaced housing on the east side of the island; this village spans approximately 600 × 200 metres; there is a primary school, health clinic and a church. The island population was actively engaged and enthusiastic about the project, and their thoughts and suggestions were carefully considered by the project team.

## Methods

### Filariasis blood testing

The community leaders were unhappy about the idea of a randomized survey because some people might have been upset if they were excluded from participating, so a convenience sample was used. Permission could not be obtained to test children under 12 years old. EDTA blood was collected by venipuncture between the hours of 10 pm and 2 am. All samples were tested for filarial antigen by the ICT card test (AMRAD ICT, NSW, Australia) and for microfilaraemia by a Knott’s concentration test [7], using 2 ml of blood. The total microfilaria count divided by two was used to determine microfilaria intensity per millilitre of blood.

### Clinical survey

Local health workers believed that there were no cases of lymphoedema or elephantiasis cases on the island, but experience in other places has shown that sufferers may remain in their houses due to disability or embarrassment about being seen in public. To avoid the possibility of cases being missed, a house by house search was done. Due to the absence of a male medical officer, it was impossible to do medical examinations to check for hydrocoele, but local health workers agreed with the suggestion of the JCU female social scientist that a de facto survey could be done by asking wives if their husband had a hydrocoele.

### Entomology

Satawal Island was sampled for mosquitoes over a 4-day period in late January 2003. CDC light traps and Gravid traps were set in and around homes as well as in garden and forest areas. Larval collections were made from water containers, natural habitats, such as halved coconuts, drainage ditches through garden patches and forest pools of fresh water used as wells. Landing and biting adults were collected from these locations as opportunities presented. Analysis of adults took place several days after mosquitoes had been caught. Dissections for larvae were possible in the FSM hospital laboratory. Larvae were observed in live preparations without the 10-day staining/destaining/dissection procedure.

### Interviews

Local adult residents and chiefs of both genders were interviewed for their opinions and attitudes about LF in the past and current.

### Statistical analysis

Significance of differences in categorical test results by gender and age and antigenaemia compared to microfilaraemia were determined using Pearson’s chi-squared test. Significance of differences in quantitative results by gender and age were determined using Student’s *t* test on the log Mf counts. A *p* value of <0.05 was considered to be significant.

## Results

### Prevalence of filariasis in tested population

Blood was collected from 253 participants (104 males and 149 females). Ages ranged from early teens to mid-80s. Overall, 96 participants (38%) showed antigenaemia by the ICT card test and 55 participants (22%) were found to have circulating microfilaria in Knott’s concentration test (*p* < 0.001). ICT detected more infections than Knott’s concentration in all age and gender groups. Men had a significantly higher percentage prevalence of ICT positive results than women (53 vs 28%; *p* < 0.001); this difference was maintained in Knott’s concentration results (37 vs 11%; *p* < 0.001). Overall prevalence of filaraemia was higher in subjects ≤25 than in those >25 years of age Knott’s concentration test (29 vs 18%; *p* = 0.048) (Table 1). No significant difference in antigenaemia by filariasis ICT test was observed between the two groups (45 vs 34%, *p* = 0.08).

**Table 1:** Results of filariasis survey on Satawal Island, January 2003 (*n* = 253)

Gender	Age group	Prevalence		Mf density	Mf count
		ICT	Knotts	Geometric mean^a^	Range^a^
		% (*n*)	% (*n*)	Mf/mL	Mf/mL
Males	≤15 years old (*n* = 16)	56 (9)	31 (5)	21	5–86
	16–20 years old (*n* = 10)	40 (4)	30 (3)	16	6–26
	21–25 years old (*n* = 12)	75 (9)	66 (8)	24	7–204
	>25 years old (*n* = 66)	50 (33)	33 (22)	51	10–866
	Total all ages (*n* = 104)	53 (55)	37 (38)	36	5–866
Females	≤15 years old (*n* = 14)	50 (7)	36 (5)	26	7–620
	16–20 years old (*n* = 20)	50 (10)	25 (5)	4	2–11
	21–25 years old (*n* = 19)	11 (2)	0 (0)	0	n/a
	>25 years old (*n* = 96)	23 (22)	7 (7)	13	4–204
	Total all ages (*n* = 149)	28 (41)	11 (17)	11	2–620
Combined (male + female)	≤15 years old (*n* = 30)	53 (16)	33 (10)	23	5–620
	16–20 years old (*n* = 30)	47 (14)	27 (8)	7	2–26
	21–25 years old (*n* = 31)	35 (11)	29 (26)	24	7–204
	>25 years old (*n* = 162)	34 (55)	18 (29)	37	4–866
	Total all ages (*n* = 253)	38 (96)	22 (55)	25	2–866

*ICT* antigen test, *Knotts* Knott’s concentration technique, *Mf* microfilaria, *n* number examined, *n/a* not applicable

^a^Mf pos cases only, as determined by Knott’s concentration technique

### Density of filaraemia in tested population

Microfilaria density ranged from 2 to 866, with a mean of 87, geometric mean of 25 and median of 18 microfilaria per millilitre of blood in Mf positive cases (Table 1). When comparing positive Mf individuals, men had significantly higher geometric mean Mf density (36) than women (11) (*t* = 2.80, *p* = 0.0035). Geometric mean Mf density in those positive were however significantly higher in older (37 Mf/mL) than in younger (16 Mf/mL) individuals (*t* = −2.14, *p* = 0.0185).

### Clinical survey

All houses on the island were surveyed for elephantiasis and questioned regarding hydrocoele. No cases of elephantiasis were found, and no wives reported their husbands having hydrocoele. Unfortunately, the specific number of houses and individuals interviewed was not retained, and these data are not available.

MDA, comprising the WHO-recommended regimen of a single oral dose of DEC (6 mg/kg body weight) and albendazole (400 mg regardless of weight) under direct observation was given immediately after the blood was taken for testing. A program of annual MDA was then started in Satawal.

### Entomology

Adult mosquitoes were collected on Satawal Island near forest pools adjacent to gardens, namely, *Verrallina* (*Verrallina*) sp. and *Aedes* (*Stegomyia*) *scutellaris scutellaris*. The specimens from gravid traps were identified as *Ae* (*Stg*) *scutellaris scutellaris* and *Culex quinquefasciatus*. Larval specimens from forest pools were identified as *Cx annulirostris*, while larvae from forest coconuts were identified as *Ae* (*Stg*) *scutellaris* gp.

Dissections were made before accurate identification of adults could take place; however, mosquitoes were sorted into those most likely to be *Aedes* (*Stg*) and those that may have been *Verrallina* or *Culex* (both dark varieties with few leg markings). No *Aedes* (*Stg*) *scutellaris* gp were positive for L1–3 stages. Three of nine probable *Verrallina* sp. (but possibly *Culex quinquefasciatus*/*annulirostris*) were positive for L1–3 larval stages of *W. bancrofti* by microscopy.

## Discussion

There is no doubt that the transmission of filariasis on Satawal Island was still ongoing in 2003, as demonstrated by the high prevalence of infection in both humans and mosquitoes (though only a small number of adult mosquitoes were tested). There were numerous mosquito breeding sites, and there was close interaction between mosquitoes and people. This was evidenced by the differing filarial prevalences between males and females. While such differences have been reported in many places, the local people on Satawal provided a very sensible suggestion as to why more males than females were infected. Males tend to socialize outdoors in the late afternoon and early evening during the peak mosquito biting time, whereas the women are more likely to be cooking around smoky fires and less likely to be bitten.

No cases of lymphoedema or elephantiasis were found on the island in the 2003 survey. Neither condition was not reported as having ever been seen there in the living memory of even the very old. Anecdotally, some of the older people believed that hydrocoele was historically present, though no cases of hydrocoele were identified in this 2003 survey.

The prevalence of lymphoedema and elephantiasis is usually related to the intensity of transmission but is mitigated by good hygiene. Hygiene was reasonable on Satawal, but if filariasis has been present for a long time, it was surprising that some cases have not occurred. Therefore, there was a strong possibility that this focus of infection represented a relatively new incursion, and although the prevalence was high, there had not been a time to cause a lot of chronic pathology. This apparent re-emergence of transmission toward large-scale infection in an isolated area has significant implications for other regions where filariasis has been eliminated; in that, it indicated that a return of infection is quite possible in such places without regular monitoring in the years immediately post-elimination. The theory of relatively recent introduction of the disease is supported by the relatively high infection rate in younger individuals. When compared to other studies, the density of microfilaraemia in the Satawalese was low in comparison to the prevalence of disease. Kimura et al.’s 1994 study in Chuuk showed an LF prevalence of 2.6% and (geometric) microfilaria density of 185/mL^5^. In this 2003 Satawal study, a 22% prevalence was described, but only 25 Mf/mL geometric density. In Sri Lanka, where *Culex quinquefasciatus* transmit filariasis, a prevalence of 4.4% and microfilaria density of 343/ml were reported [8]. It may be the case that intense transmission of LF occurred at a time when the mosquito population was extremely high, leading to widespread, very low density infections in Satawal.

Upon further questioning, several locals revealed more information that qualified the previous offerings. In 1993–1994, Satawal Island experienced a surge in mosquitoes the like of which none of those interviewed had ever experienced before. This may have resulted in the disease becoming uncommonly more prevalent. Apparently, *C. quinquefasciatus* and *C. annulirostris* (brown mosquitoes) were always a big problem in the past (prior to 1993–1994) with significant nuisance populations. The mosquito population became so great that locals found themselves covered with biting mosquitoes during peak biting periods in the mornings and evenings.

Several male and female locals said that one used to be able to kill dozens of mosquitoes in a single-hand smack and that the skin would turn dark with so many mosquitoes biting. All agreed that they had never experienced a mosquito problem this large at any time or on any other island in the past. The situation became so intense that the chiefs met to determine a solution. They decided that all the locals would prepare palm fronds by drying so that they could be set alight and used to burn the mosquitoes. For many days, especially during early evening peak biting times, everyone on the island was involved in a community effort to wipe out mosquitoes. The palm fronds were set alight and were waved through the air by adults, children, men and women. Some informants reported being able to see hordes of mosquitoes falling from the air with their wings singed after a flaming frond had passed by. Breeding sites were targeted as were communal areas and areas surrounding residences. This activity continued for an undetermined period (some said many days, some said less than weeks—no one was sure) until the mosquitoes ceased to be a nuisance. All informants reported that the brown mosquito population had been low ever since.

## Conclusions

The findings on Satawal had important ramifications for the rest of FSM, and indeed others parts of the Pacific. Firstly, this survey showed that isolated high-intensity foci of filariasis infection do occur and can remain unrecognized in areas thought to be close to elimination unless detailed surveys are undertaken, and if this focus represented a new incursion, it shows how filariasis might move into a previously uninfected area or re-emerge in a previously controlled area due to travel or migration, a fact with implications for the current worldwide eradication efforts.

## Abbreviations

CDC: Centers for Disease Control and Prevention
DEC: Diethylcarbamazine
FSM: Federated States of Micronesia
ICT: Immunochromatographic test
LF: Lymphatic filariasis
MDA: Mass drug administration
Mf: Microfilaria
